# The self-assembled nanoparticle-based trimeric RBD mRNA vaccine elicits robust and durable protective immunity against SARS-CoV-2 in mice

**DOI:** 10.1038/s41392-021-00750-w

**Published:** 2021-09-09

**Authors:** Wenqiang Sun, Lihong He, He Zhang, Xiaodong Tian, Zhihua Bai, Lei Sun, Limin Yang, Xiaojuan Jia, Yuhai Bi, Tingrong Luo, Gong Cheng, Wenhui Fan, Wenjun Liu, Jing Li

**Affiliations:** 1 CAS Key Laboratory of Pathogenic Microbiology and Immunology, Institute of Microbiology, Chinese Academy of Sciences, Beijing, China; 2 Institute of Infectious Diseases, Shenzhen Bay Laboratory, Shenzhen, Guangdong China; 3 State Key Laboratory for Conservation and Utilization of Subtropical Agro-Bioresources & Laboratory of Animal Infectious Diseases, College of Animal Sciences and Veterinary Medicine, Guangxi University, Nanning, Guangxi China; 4 University of Chinese Academy of Sciences, Beijing, China; 5 Tsinghua-Peking Center for Life Sciences, School of Medicine, Tsinghua University, Beijing, China; 6 Institute of Microbiology, Center for Biosafety Mega-Science, Chinese Academy of Sciences, Beijing, China

**Keywords:** Vaccines, Infectious diseases

## Abstract

As COVID-19 continues to spread rapidly worldwide and variants continue to emerge, the development and deployment of safe and effective vaccines are urgently needed. Here, we developed an mRNA vaccine based on the trimeric receptor-binding domain (RBD) of the SARS-CoV-2 spike (S) protein fused to ferritin-formed nanoparticles (TF-RBD). Compared to the trimeric form of the RBD mRNA vaccine (T-RBD), TF-RBD delivered intramuscularly elicited robust and durable humoral immunity as well as a Th1-biased cellular response. After further challenge with live SARS-CoV-2, immunization with a two-shot low-dose regimen of TF-RBD provided adequate protection in hACE2-transduced mice. In addition, the mRNA template of TF-RBD was easily and quickly engineered into a variant vaccine to address SARS-CoV-2 mutations. The TF-RBD multivalent vaccine produced broad-spectrum neutralizing antibodies against Alpha (B.1.1.7) and Beta (B.1.351) variants. This mRNA vaccine based on the encoded self-assembled nanoparticle-based trimer RBD provides a reference for the design of mRNA vaccines targeting SARS-CoV-2.

## Highlights

1. Nanoparticle-based trimeric RBD mRNA vaccine induces potent neutralizing antibody responses.
2. Nanoparticle-based trimeric RBD mRNA vaccine provides protection against SARS-CoV-2 infection in mice.
3. Low-dose mRNA vaccines elicit both humoral and cellular immune responses.
4. Multivalent vaccine promotes neutralizing antibodies responses against pseudoviruses.

## Introduction

Coronavirus disease 2019 (COVID-19), caused by severe acute respiratory syndrome coronavirus 2 (SARS-CoV-2), is wreaking havoc worldwide. It causes severe respiratory disease and the failure of numerous organs, thereby posing a substantial threat to human health.^1^ As of May 2020, the total number of COVID-19 cases had exceeded 160 million worldwide, with more than 3 million confirmed deaths, according to the World Health Organization (https://www.who.int/). Safe and effective prophylactic vaccines are urgently needed to combat the pandemic, particularly in response to typical rapid strain mutations.

SARS-CoV-2 belongs to the coronavirus family and is an RNA virus encoding enveloped and single-stranded RNA. SARS-CoV-2, along with SARS-CoV and MERS-CoV, belong to the genus β-coronavirus. Structural proteins of the virion include the spike (S) protein, nucleoprotein (N), envelope (E) protein, and membrane (M) protein.^2–4^ At present, many vaccines have been developed, all of which target structural proteins, and the S protein is the most commonly used. The S protein is comprised of two protein subunits (S1 and S2), where S1 is responsible for binding to the ACE2 receptor and S2 is responsible for fusion with the cell membrane.^5,6^ In general, blocking the binding of S to the receptor prevents viral entry into host cells. Two COVID-19 mRNA vaccines (BNT162b2, Pfizer-BioNTech; mRNA-1273, Moderna), which were approved by the Food and Drug Administration (FDA) for Emergency Use Authorization (EUA), are based on nucleoside-modified mRNAs encoding the stabilized prefusion S glycoprotein. The current data show high neutralizing antibody titers and effective protection rates (>90%) in human clinical trials.^7–10^ The same selection of the S protein as an antigen has been reported in adenovirus vector vaccines, DNA vaccines, and subunit vaccines.^11–15^ Overall, the use of S proteins for vaccines has been verified to produce effective immune protection in animal models and human clinical trials.

The receptor-binding domain (RBD) comprises a portion of the S1 subunit and is the smallest domain that directly binds to the corresponding receptor.^12^ Both mRNA and subunit vaccines based on the monomer or dimer RBD as the antigen have been studied.^16–18^ Furthermore, the trimeric RBD subunit vaccine of our previous work has been validated in non-human primates.^19^ Although antibody-dependent enhancement (ADE) of infection has not been reported for the existing COVID-19 vaccines, it has been reported with SARS-CoV and MERS-CoV.^20–24^ Therefore, selecting the RBD as the vaccine antigen can minimize the ADE effect and thus increase vaccine safety.^25–27^ The RBD monomer is very small in size, has a poor immunogenicity, and is not easily captured by the immune system. Therefore, neither the monomer nor the dimer meets the requirements.^28–30^ Two subunit vaccine studies reported improvement of the RBD immunogenicity by cross-linking the RBD monomer to nanoparticles, using ferritin as a scaffold.^30,31^ Excitingly, compared with monomeric RBD, nanoparticle RBD can produce stronger humoral and cellular immune responses. However, monomeric RBD binds to only nanoparticles and cannot maintain a stable natural trimer conformation. Moreover, the production process is complex, requiring separate expression and purification processes for ferritin and the antigen and further purification after cross-linking, and the RBD proteins are not guaranteed to be fully loaded onto the nanoparticles. Furthermore, subunit vaccines based on the full-length S protein or RBD fused with ferritin have also been reported^32,33^; however, some deficiencies remain regarding nanoparticle-based subunit vaccine technology. Importantly, the extension of this technology has provided an additional platform for the development of mRNA vaccines. The mRNA vaccine platform is substantially advantageous in terms of the potency of the delivered antigen, which is the most natural modification of the viral protein encoded by the host.^34^ Therefore, it is necessary to explore whether an mRNA vaccine encoding the nanoparticle-based trimeric RBD has good immune effects.

SARS-CoV-2 is well-known to mutate rapidly, with the main variants Alpha (B.1.1.7), Beta (B.1.351), Gamma (P.1), Iota (B.1.526), B.1.427/B.1.429/CAL.20C, Delta (B.1.617.2), and Lambda (C.37) being reported thus far.^35–38^ In particular, the Alpha and Beta lineages have been studied and reported most frequently. Studies have shown that the RBD domain of the Alpha mutant strain carries an N501Y mutation, which increases the binding capacity of ACE2 to increase its transmissibility; however, the serum neutralization titers are not significantly decreased in vaccinated or recovered patients.^39–41^ Importantly, variant Beta, with three mutations in the RBD domain (K417N, E484K, and N501Y), was shown to alter antigenicity and to partially evade immune responses to past infections or existing vaccines.^41–44^ Because strain mutations pose a substantial challenge for existing vaccines, a broad-spectrum vaccine is urgently needed. Notably, the current approved vaccines improve our understanding of the key advantages of mRNA technology.^9,45,46^ The wild-type mRNA vaccine can be rapidly transformed into a variant vaccine by mutating the nucleotide sequence of the mRNA template, representing an uncomplicated approach that is currently being implemented in the mRNA vaccine industry.

Previous studies have attempted to develop mRNA vaccines targeting the Ebola, Zika, and influenza viruses and cancer and to assess their abilities to induce effective protective immune responses and their safety.^45,47–49^ To develop a safe, effective and long-term vaccine to move past the bottleneck regarding existing vaccines, we extended the mRNA technology to this study. Here, we evaluated the humoral and cellular immune responses elicited by a nanoparticle-based trimeric RBD mRNA vaccine candidate in mice, investigated the kinetics of the multivalent vaccine against variant pseudoviruses, and demonstrated the production of robust and durable immune responses and strong protection in hACE2-transduced mice challenged with SARS-CoV-2.

## Results

### Characterization of the mRNA vaccine candidates T-RBD and TF-RBD

**Fig. 1:**
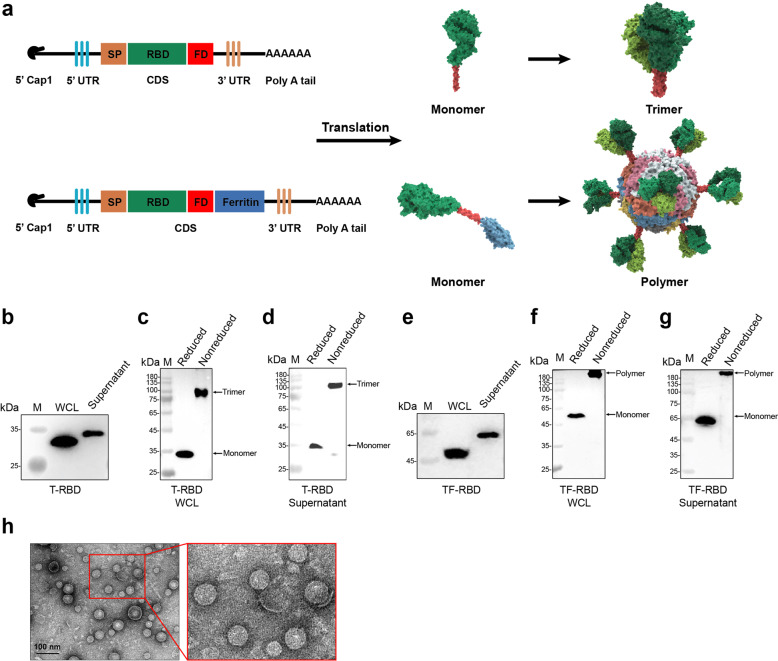
Trimeric form of SARS-CoV-2 RBD (T-RBD) and the self-assembled ferritin nanoparticle form of SARS-CoV-2 RBD (TF-RBD). **a** Schematics of the T-RBD and TF-RBD designs. The RBD (R391-D541) of the SARS-CoV-2 spike (S) protein containing the N-terminal tPA signal peptide (SP) and the C-terminal foldon (FD) trimer tag or linked to ferritin (N96 mutation; increase protein secretion) gene sequences of *Helicobacter pylori* was synthesized in a DNA plasmid by codon optimization. The modified mRNA was synthesized by the in vitro transcription (IVT) of a linearized DNA plasmid. **b** The expression of the T-RBD protein in the whole-cell lysate (WCL) and supernatant was analyzed by western blot. **c**, **d** Western blot profiles of T-RBD protein expression under reduced and nonreduced conditions. **e** The expression of the TF-RBD protein in the WCLs and concentrated supernatant was analyzed by western blot. **f**, **g** Western blot profiles of TF-RBD protein expression under reduced and nonreduced conditions. **h** Transmission electron microscopy (TEM) images showing nanoparticles formed by TF-RBD expressed in the supernatant of HEK293T cells (scale bar, 100 nm)

To maintain the natural trimer conformation and enhance the immunogenicity of the SARS-CoV-2 RBD, we designed two trimer conformation antigens, the trimeric form of the SARS-CoV-2 RBD (T-RBD) and a trimeric form of the RBD displayed by *Helicobacter pylori* ferritin (TF-RBD). The gene sequences of the two antigens were inserted into an mRNA transcription plasmid carrying the T7 promoter. Subsequently, the mRNAs of T-RBD and TF-RBD were transcribed by a linearized mRNA transcription plasmid with the modified nucleoside N1-methylpseudouridine, 5′ Cap1, and 3′poly A tail (Fig. 1a). To verify their protein translation abilities, the two mRNAs were transfected into HEK293T cells. Western blot analysis revealed that T-RBD was partially secreted into the cell supernatant, while some remained in the cytoplasm. The molecular weight of the secreted T-RBD was significantly higher than that of intracellular T-RBD, clearly indicating that the secreted T-RBD had been glycosylated (Fig. 1b). Because the SARS-CoV-2 S protein itself is a glycoprotein, glycosylation is necessary for the T-RBD antigen. Moreover, the T-RBD protein formed a trimer both extracellularly and intracellularly, with a molecular weight difference of exactly threefold (Fig. 1c, d). Similar expected results were obtained in TF-RBD for the TF-RBD mRNA (Fig. 1e). In the natural state, ferritin self-assembles into a 24-subunit protein-based nanoparticle. The N-termini of the three ferritin monomers form a vertex, which facilitates the formation of a fusion protein trimer on the vertex. Here, T-RBD was added to the N-terminus of ferritin using a flexible linker to yield a trimeric RBD on ferritin nanoparticles and thereby enhance immunogenicity. The reduced and nonreduced western blot results showed that TF-RBD was displayed in polymeric states both extracellularly and intracellularly (Fig. 1f, g). TEM analysis of the concentrated TF-RBD supernatant revealed nanoparticles with a diameter of 30 nm (Fig. 1h).

**Fig. 2:**
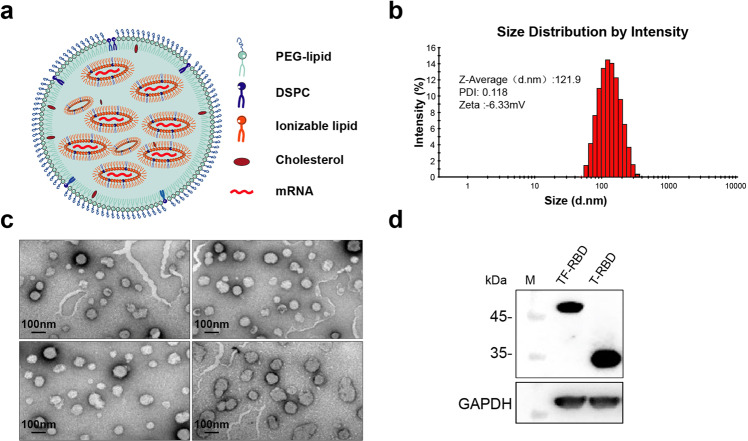
Formulation and physical properties of liposomal nanoparticles (LNPs) for codelivery. **a** LNPs were synthesized from PEG-lipid (1.5 mol/mol), DSPC (10 mol/mol), ionizable lipid (50 mol/mol), and cholesterol (38.5 mol/mol) at a specific ratio. **b** The particle size, uniformity, and zeta potential of the LNPs were measured by a Malvern particle size instrument. **c** TEM images of the LNPs (scale bar, 100 nm). **d** The mRNA expression of the LNPs (T-RBD and TF-RBD proteins) in HEK293T cells was analyzed by western blot

To prevent nuclease degradation and promote efficient expression in vivo, we encapsulated the mRNAs of T-RBD and TF-RBD into LNPs (Fig. 2a). To evaluate the physical properties of the LNPs, dynamic light scattering, and TEM analyses were performed. The dynamic light scattering of LNPs revealed a mean particle size of 121.9 nm, with a narrow polydispersity index (PDI) of 0.118 (Fig. 2b). In addition, the TEM results revealed complete nanoparticle morphology, and the size was consistent with that determined by dynamic light scattering (Fig. 2c). The zeta potential measurement of the LNPs was −6.33 mV in PBS (pH 7.2). To assess the delivery effect of LNPs in vitro, LNPs were added to precultured HEK293T cells. The western blot results showed that T-RBD and TF-RBD were both high expressed in HEK293T cells (Fig. 2d).

### The TF-RBD mRNA vaccine candidate induced strong humoral and cellular immune responses in vivo

**Fig. 3:**
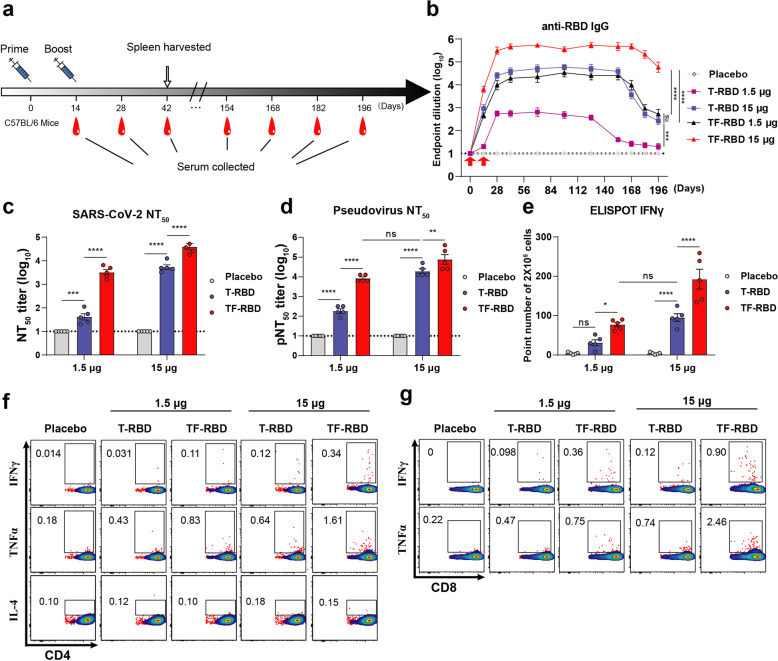
Screening and evaluation of the T-RBD or TF-RBD mRNA vaccine candidates. **a** Immune procedures for evaluating the T-RBD or TF-RBD mRNA vaccine candidates. Groups of 6- to 8-week-old female naive C57BL/6 mice (*n* = 10) were vaccinated with two doses (1.5 μg, low; 15 μg, high) of the mRNA LNPs at 2-week intervals. Blood collection and spleen extraction were performed at the time points shown after immunization. **b** ELISA revealed the RBD-specific IgG titer of SARS-CoV-2. Serum samples were collected after each immunization event and used for the detection of IgG to evaluate the humoral response dynamics. A placebo formulation was given as the control. The red arrows indicate the immunization time point. **c**, **d** Neutralization assays of live SARS-CoV-2 in (**c**) and the SARS-CoV-2 pseudovirus in (**d**). **e** Protein-specific T cell ELISPOT assay results. Spleen cells (2 × 10^5^) were stimulated with the S RBD protein at a final concentration of 10 μg/mL. **f**, **g** Protein-specific ICS assays. Spleens were harvested at 28 days after booster immunization, and the cells were stimulated with the S RBD protein at a final concentration of 10 μg/mL. The proportions of CD4^+^ protein-specific T cells in (**f**) and CD8^+^ protein-specific T cells in (**g**) are shown. The data are shown as the mean ± standard error of the mean (SEM). The dotted horizontal lines indicate the limits of quantification for ELISA and NT_50_ titers. P values were determined by one-way ANOVA (ns, *p* > 0.05; *p < 0.05; **p < 0.01; ***p < 0.001; ****p < 0.0001). See also Figures S1–S4

To evaluate humoral and cellular immune responses, mice were immunized intramuscularly twice with a high (15 μg) or low (1.5 μg) dose or with a placebo, and samples were collected at specific points (Fig. 3a). The antibody levels in all of the immunized groups of mice were significantly increased after booster immunization. Mice in the TF-RBD 15-μg dose group exhibited the highest antibody levels, which were significantly higher than those observed in other group, and the antibody titers remained high level for at least 6.5 months. Interestingly, the antibody levels did not significantly differ between the 15-μg dose T-RBD group and the 1.5-μg dose TF-RBD group (Fig. 3b, Supplementary Figure 1).

The live SARS-CoV-2 NT_50_ values were highest in the TF-RBD 15-μg dose group, which exhibited significantly higher values than the other groups. There was no significant difference in the values between the T-RBD 15-μg dose group and the TF-RBD 1.5-μg dose group. In addition, the low dose of T-RBD induced the lowest NT_50_ values, consistent with those determined by the pseudovirus neutralization (PsVN) assay (Fig. 3c, d). We also evaluated whether the neutralizing antibody titers were associated with the ELISA titers, revealing a positive correlation between the two for both live SARS-CoV-2 (*r* = 0.342, *P* < 0.0001) and the pseudovirus (*r* = 0.8797, *P* < 0.0001) (Supplementary Figure 2). Importantly, these results suggested that the selection of the RBD as the immunogen produces higher levels of neutralizing antibodies than those in the sera of convalescent patients.^50^ In general, the TF-RBD strategy induced higher levels of humoral immunity. The ELISPOT results indicated that IFNγ secretion by T lymphocytes was significantly higher in the 15-μg dose TF-RBD group than in the other groups, and the difference between the 15-μg dose T-RBD group and the 1.5-μg dose TF-RBD group was not significant. The secretion levels of IFNγ were the lowest in the T-RBD 1.5-μg dose group (Supplementary Figure 3). Furthermore, ICS assays were performed to quantify the proportions of protein-specific IFNγ and TNFα in Th1 CD4^+^ and CD8^+^ T cells and IL-4 in Th2 CD4^+^ (Fig. 3e–g, Supplementary Figure 4). In general, both vaccines were shown to stimulate cellular immune response and mainly stimulated the Th1-biased response, with TF-RBD being the strongest; Notably, no Th2-related vaccine-associated enhanced diseases (VAEDs) were found. In summary, the humoral and cellular immune responses induced by TF-RBD were stronger than those induced by T-RBD. Therefore, TF-RBD represents an ideal candidate for SARS-CoV-2 vaccine development and has a stronger activation ability than T-RBD.

### Low doses of the TF-RBD mRNA vaccine candidate provide complete protection in mice

**Fig. 4:**
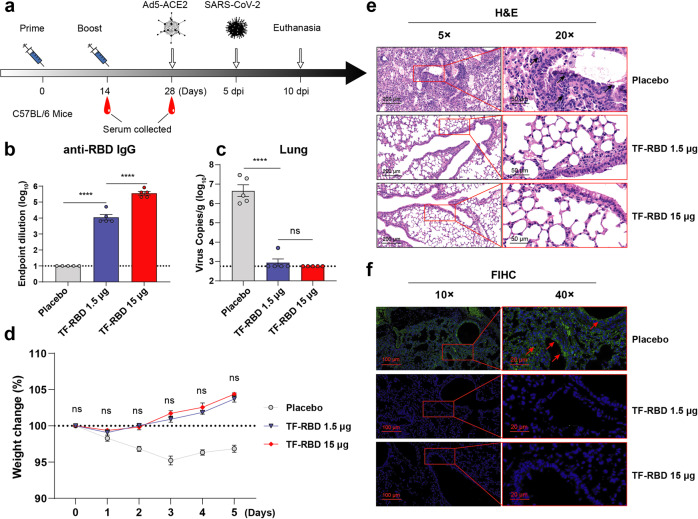
Evaluation of the immune protection provided by the TF-RBD mRNA vaccine candidate during challenge in vivo. **a** Immunization and challenge procedures for the 1.5 and 15 μg TF-RBD mRNA vaccine candidates in mice. Naive 6- to 8-week-old female naive C57BL/6 mice (*n* = 5) were administered two doses of the vaccines via the intramuscular route. The mice were infected with Ad5-ACE2 and subsequently challenged with live SARS-CoV-2. Blood collection was performed at the indicated time points after immunization (14 days after prime immunization and 14 days after booster immunization). **b** The anti-RBD-specific IgG antibody titers in the vaccine and placebo groups were determined by ELISA. **c** The viral RNA loads (copies/g) in the mouse lungs were measured. **d** The body weights of the mice were monitored and recorded for 5 consecutive days after the challenge. The mice were euthanized after observation. **e** Hematoxylin and eosin (H&E) staining to assess pathological changes in the lungs of mice at 5 dpi. The black arrows indicate the infiltrating inflame cells in the alveolar space (scale bar, left 5 × 200 μm, right 20 × 50 μm). **f** Fluorescence immunohistochemistry (FIHC) staining images of mouse lungs at 5 dpi. The red arrows show the sections incubated with the anti-SARS-CoV-2 S protein antibody, and the blue fluorescence indicates the cell nuclei locations as determined by DAPI staining (scale bar, left 10 × 100 μm, right 40 × 20 μm). The dotted horizontal lines indicate the limits of quantification for the ELISA and viral RNA load data. The data are shown as the mean ± SEM. *P* values were determined by one-way ANOVA (ns, *p* > 0.05; *p < 0.05; **p < 0.01; ***p < 0.001; ****p < 0.0001)

To further explore the optimal dose of the TF-RBD mRNA vaccine candidate for protection efficacy in vivo, a SARS-CoV-2 challenge experiment was performed in immunized mice (Fig. 4a). Serum was collected 2 weeks after booster immunization to detect the anti-RBD-specific IgG antibody levels. The results showed that the 15- and 1.5-μg dose groups exhibited mean titers of 10^5.5^ and 10^4.1^, respectively (Fig. 4b), which were similar to previous results. The SARS-CoV-2 challenge experiment was carried out 5 days after transduction of the human ACE2 gene into mouse lungs by an adenovirus vector. Analyses of the lung viral RNA load revealed no virus in the 15-μg dose group, while no viral RNA was detected in all but one mouse in the 1.5-μg dose group. In contrast, more viral RNA was detected in the mice immunized with the placebo (Fig. 4c). The body weight changes were not significantly different between the 1.5- and 15-μg dose groups, but rapid weight loss was observed in the placebo group (Fig. 4d). To further observe the pathological changes in the lungs after infection, paraffin-embedded tissue sections were subjected to H&E staining. No significant pathological features were observed in the lungs of mice in the 1.5- and 15-μg dose groups, while large amount of infiltrating inflame cells were observed in the alveolar space of mice immunized with the placebo (Fig. 4e). In addition, FIHC staining revealed no viral replication of the anti-S protein antibody in the lungs of mice in the 1.5- and 15-μg dose groups, which was significantly different from that in the lungs of mice receiving the placebo (Fig. 4f). Interestingly, in the one mouse exhibiting viral RNA in the 1.5-μg dose group, nucleic acid from the dead virus could have feasibly entered through the nasal passage, which is in accordance with previous observations of respiratory infection.^51^ These data demonstrated that a two-dose immunization strategy with a low dose of the TF-RBD mRNA vaccine candidate was sufficient to produce a protective immune response against SARS-CoV-2 in mice.

### The TF-RBD-triCoV vaccine elicits cross-neutralizing antibody responses

**Fig. 5:**
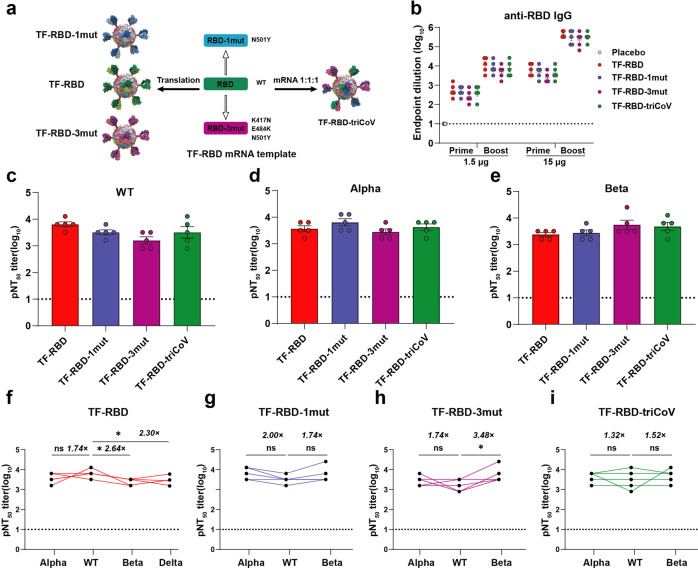
The trivalent TF-RBD (TF-RBD-triCoV) mRNA vaccine candidate enhances cross-neutralization. **a** Schematic diagram of the TF-RBD-triCoV mRNA vaccine. TF-RBD-triCoV contains TF-RBD, TF-RBD-1mut (N501Y), and TF-RBD-3mut (K417N, E484K, N501Y) mRNA with mass ratio of 1:1:1. **b** Groups of 6- to 8-week-old female naive C57BL/6 mice (*n* = 5) were vaccinated by secondary immunization at 2-week intervals. The RBD-specific IgG titer of SARS-CoV-2 was revealed by ELISA. **c**–**e** Pseudovirus neutralization assay of the 1.5-μg dose group shows the NT_50_ values for the WT virus (**c**), variant Alpha (**d**), and variant Beta (**e**). **f**–**i** Changes in the serum neutralization titers of each vaccine in the 1.5-μg dose group against different pseudoviruses. The following cross-NAb titers are demonstrated: TF-RBD (**f**), TF-RBD-1mut (**g**), TF-RBD-3mut (**h**), and TF-RBD-triCoV (**i**). The dotted horizontal lines indicate the limits of quantification for the ELISA and NAb titers. The data are shown as the mean ± SEM. *P* values were determined by one-way ANOVA (ns, *p* > 0.05; *p < 0.05; **p < 0.01; ***p < 0.001; ****p < 0.0001). See also Figures S5 and S6

In response to the current SARS-CoV-2 mutations, TF-RBD-1mut (N501Y) and TF-RBD-3mut (K417N, E484K, N501Y) were designed based on the mRNA template of TF-RBD. In addition, a trivalent mRNA vaccine candidate, TF-RBD-triCoV, was produced with TF-RBD, TF-RBD-1mut, and TF-RBD-3mut mRNA at a ratio of 1:1:1 (Fig. 5a). Since the ferritin is shared by these RBDs, they can be assembled into nanoparticles containing various types of RBDs at random. The Co-IP assay showed that all three types of RBD in TF-RBD-triCoV were expressed effectively (Supplementary Figure 5). Primary female C57BL/6 mice (*n* = 5) aged 6–8 weeks were prime-boost immunized with high (15 μg) and low (1.5 μg) doses at an interval of 2 weeks, and their sera were collected for detection of the anti-RBD-specific IgG antibody. The trend was consistent with that in our previous data, with slight reductions in the antibody titers in the variant and TF-RBD-triCoV mRNA vaccine candidate groups. These reductions may have reduced the ability of the antibodies produced by the variant mRNA vaccine candidate to bind to the WT RBD protein (Fig. 5b). To verify the neutralization ability of the candidate vaccine against different variants in vitro, cross-neutralizing pseudovirus assays were performed in the low-dose group. The vaccine group matched with the pseudovirus exhibited the highest neutralization titer. Importantly, the TF-RBD-triCoV group exhibited similar neutralizing antibody titers for different pseudovirus variants, while the other vaccine groups exhibited decreasing trends for mismatched variants (Fig. 5c–e). Moreover, we further statistically analyzed the neutralization titers of each vaccine against pseudoviruses, revealing that the TF-RBD-induced neutralization antibody titers against Alpha pseudovirus were reduced by 1.74-fold, while those against the Beta pseudovirus were significantly reduced by 2.64-fold compared with WT. Furthermore, TF-RBD also induced neutralization antibody titer by 2.30-fold significant reduction against Delta pseudovirus (Fig. 5f). Compared with that against WT pseudoviruses, the neutralization titer induced by TF-RBD-1mut for the Alpha pseudoviruses was increased by 2.00-fold, while that of Beta pseudoviruses was reduced by 1.74-fold (Fig. 5g). In addition, compared with that of WT pseudoviruses, the neutralization titer induced by TF-RBD-3mut was reduced by 1.74-fold for the Alpha virus and significantly reduced by 3.48-fold for Beta pseudoviruses (Fig. 5h). Encouragingly, the effects of TF-RBD-triCoV were not significantly different among any of the three pseudoviruses tested (Fig. 5i). The TF-RBD-triCoV multivalent vaccine was shown to produce broad-spectrum neutralizing antibodies against different pseudoviruses in mice. Moreover, we further investigated the humoral immunity of the mice pre-immunized with two doses of TF-RBD then vaccinated with a third dose of TF-RBD-triCoV at day 196 (Supplementary Figure 6a). The ELISA results showed that the anti-RBD IgG (Supplementary Figure 6b) and pNT_50_ (Supplementary Figure 6c–e) titers at day 210 were increased and were higher than that at day 196 again different pseudoviruses. In addition, the pNT_50_ values also revealed an increase in the cross-neutralization titer after the third vaccination of TF-RBD-triCoV (Supplementary Figure 6f, g). The SARS-CoV-2 mutation is ongoing, and a third dose may be inevitable. This TF-RBD-triCoV multivalent vaccine reboost experiment provided a reference for the clinical application of the vaccine. In summary, these data suggest that the TF-RBD-triCoV mRNA vaccine can produce a broad-spectrum protection against different pseudoviruses by promoting the production of cross-neutralizing antibodies.

## Discussion

As COVID-19 continues to spread worldwide, a safe and effective SARS-CoV-2 vaccine is urgently needed. In this study, we designed two mRNA vaccine candidates that were fused with the trimeric RBD with (TF-RBD) and without (T-RBD) ferritin.

We analyzed their elicited humoral and cellular immune responses and their protective efficacies against live SARS-CoV-2 challenge in immunized mice. As a result, the TF-RBD vaccine was shown to elicit a stronger response of RBD-specific antibodies and neutralizing antibodies and to induce higher numbers of Th1 CD4^+^ and CD8^+^ T cells as a cellular immune response. In general, the over-activation of Th2 T cells led to Th2-related vaccine-associated enhanced diseases (VAEDs), but IL-4- related Th2 T cell responses have not been identified in our vaccine.^52,53^ This immune response was ~10-fold stronger than that elicited by the T-RBD vaccine. Mechanistically, ferritin nanoparticles significantly enhance the immunogenicity of the trimeric RBD because the nanoparticle antigens are more easily captured and presented by dendritic cells and macrophages.^54^ Similar ferritin subunit vaccines are being simultaneously reported.^30–33^ Of those, some monomer RBDs are linked to ferritin nanoparticles by tenon-like structures, and some are expressed by fusion to display the full-length S protein and RBD domain on the nanoparticle surface. A comparison of RBD-specific IgG titers showed that our results (10^5.5^ at the 15 μg dose) are consistent with or higher than those previously reported. In contrast, the mRNA vaccine platform can advantageously produce RBD proteins with better immunogenicity in a natural trimer conformation. Since antigenic proteins are produced by the immunized host itself, RBD proteins have better protein modifications than antigenic proteins of subunit vaccine.^55^

Furthermore, monomer RBD mRNA vaccines, which also produced high anti-RBD antibody titers, have been reported.^18,51^ Compared with these vaccines, the T-RBD mRNA vaccine did not produce the same high titer at the same dose, which may have been due to insufficient protein expression of the trimer RBD; however, RBD trimerization was displayed on the nanoparticle surface, which more closely represented the natural RBD conformation, and the antigenicity was substantially enhanced. Predictably, the production of more proteins after TF-RBD optimization will enhance immunity, which has been demonstrated in mice.

Two mRNA vaccines (mRNA-1273 and BNT162B2) have been approved for emergency use in human clinical immunization trials.^7,9,56–58^ These vaccines express the full-length S protein, and produced pNAb titers of ~10^4^ at the 1 μg dose and ~10^2-3^ at the 5 μg dose were observed in mice. Unlike the current strategies used to develop SARS-CoV-2 vaccines, our vaccine was specifically designed to form nanoparticles and to use the RBD domain as the antigen, and this method has undeniably been validated in multiple studies to produce similar or higher pNAb titers (10^4.1^ at the 1.5 μg dose). As a result, the use of low dose reduces the cost of vaccine production. Although the COVID-19 vaccines have not been reported to cause ADE, vaccines targeting SARS-CoV, MERS-CoV, and other animal coronaviruses have been shown to induce this phenomenon.^22,59^ Therefore, our vaccine design advantageously minimizes the ADE effects by reducing the production of nonneutralizing antibodies while ensuring adequate immunoprotective efficacy.

In response to variants of SARS-CoV-2 (Alpha and Beta), variant mRNA vaccine candidates were designed based on the TF-RBD mRNA template. The cross-PSvN assays showed that the trivalent vaccine (TF-RBD-triCoV) produced a broad spectrum of neutralization titers against different pseudoviruses. This result has also been reported in other studies on multivalent COVID-19 vaccines based on the full-length S or S1 proteins of the WT virus and Beta variant.^56,60^ Moreover, TF-RBD-triCoV was administered as a third dose after a prime-boost with TF-RBD, and the anti-RBD IgG antibody and cross-pNAb titers were significantly elevated, especially for Beta. Notably, some people have been immunized with the WT vaccine, but the neutralization antibody levels elicited by the WT vaccine against the variants are declining, especially for the Beta lineage. Multivalent vaccines are already being used against different viruses and variants, and their broad-spectrum protective effects can be demonstrated.^61–64^ Our three-dose regimen can potentially be used as a reference for combatting SARS-CoV-2 variants. Undoubtedly, a SARS-CoV-2 multivalent vaccine will be applicable for future responses to occurring variants.

Overall, our study demonstrated that the TF-RBD mRNA vaccine candidate induced robust and durable humoral and cellular immune response and that immunization at the low dose provided strong protection against SARS-CoV-2 in mice. Furthermore, the TF-RBD mRNA vaccine platform facilitates the easy and rapid design and production of multivalent vaccines targeting SARS-CoV-2 mutations. In the future, we will update this technology by performing codon optimization to increase protein expression and modify the signaling peptides to increase protein secretion. The effectiveness of vaccines depends on the immunogenicity of antigenic proteins, and mRNA vaccines are no exception. Our study provides valuable information for the development of encoding nanoparticle-based antigen mRNA vaccines, which deserve further evaluation in non-human primate and phase I–II clinical trials.

Our study does have some limitations. Although our vaccine has shown good protection against SARS-CoV-2 in mice, its safety and efficacy have not been investigated in nonprimates or humans; low-dose TF-RBD had a strong protective effect on mice, but the minimum protective dose was not determined herein; TF-RBD vaccine produced complete protection at 5 dpi, and different time points should be selected to determine the changes in viral lung load; the safety of ferritin vaccines has been widely reported, they have not yet been validated on mRNA vaccine platforms. In addition, our variant vaccine was tested with only a pseudoviral neutralization assay, and in vivo and in vitro evaluations with live SARS-CoV-2 are lacking.

## Materials and methods

### Ethics statement

All experiments involving SARS-CoV-2 strains were conducted in a biosafety level 3 (BSL3) laboratory, were approved by the Institute of Microbiology, Chinese Academy of Sciences (IMCAS), and complied with all relevant ethical regulations regarding animal research. The animals were housed and bred in specific pathogen-free (SPF) mouse facilities in compliance with ethical guidelines, and all animal experiments were approved by the Experimental Animal Ethics and Welfare Committee of IMCAS. The SARS-CoV-2 challenge experiments were performed in the animal biosafety level 3 (ABSL3) facility approved by IMCAS.

### Cell, viruses, and animals

African green monkey kidney epithelial cells (Vero E6 cells) (ATCC CRL-1586) and human embryonic kidney 293T cells (HEK293T cells) (ATCC CRL-3216) were maintained in Dulbecco’s modified Eagle’s medium (DMEM, Gibco) supplemented with 10% fetal bovine serum (FBS, Gibco) and penicillin (100 U/ml)-streptomycin (100 mg/mL) (Thermo Fisher Scientific). HEK293T cells stably expressing hACE2 (293T/hACE2) were kindly provided by Dr. Zhendong Zhao (Institute of Pathogen Biology, Chinese Academy of Medical Sciences & Peking Union Medical College). All cell lines tested negative for mycoplasma contamination. The SARS-CoV-2 virus (hCoV-19/China/CAS-B001/2020, GISAID No. EPI_ISL_514256-7) was propagated in Vero E6 cells. Female C57BL/6 mice were purchased from Beijing Vital River Animal Technology Co., Ltd. (licensed by Charles River) and housed and bred under suitable temperature and humidity conditions.

### mRNA synthesis

The open reading frame (ORF) of each mRNA was inserted between the 5′ and 3′ untranslated regions (UTRs) and had 110 poly A at the 3′ end. The modified mRNA (methylpseudourine-5′-triphosphate instead of UTP) was synthesized from a linearized DNA template by in vitro transcription using T7 RNA polymerase (NEB, USA), purified with a RNeasy Mini Kit (250) (QIAGEN, Germany), and resuspended in RNase-free water. The concentration of purified mRNA was determined by a NanoDrop microspectrophotometer (Thermo Fisher Scientific), and the purified mRNA was analyzed by agarose gel electrophoresis and stored frozen at −80 °C until use.

### Lipid nanoparticle formulation

After diluting the purified mRNA in citrate buffer (pH 4.0) to the desired concentration, the lipids ionizable lipid (Dlin-MC3-DMA), distearoylphosphatidylcholine (DSPC), cholesterol, and PEG-lipid (PEG2000-DMG) were dissolved in ethanol at a molar ratio of 50:10:38.5:1.5, and the water and lipid phases were mixed at a volumetric ratio of 3:1 with a microfluidics mixer (NanoAssemblr, Canada) to formulate lipid nanoparticles (LNPs). The LNPs were dialyzed in phosphate-buffered saline (pH 7.4) to remove ethanol for 24 h and concentrated to a desired concentration using centrifugal filters (Merck, Ireland). Finally, the LNPs were passed through a 0.22-μm filter and stored at 4 °C until use. The particle size and zeta potential of the LNPs were measured by a Malvern instrument (Malvern, England), and their encapsulation efficiency was determined by an RNA Assay Kit (Thermo Fisher Scientific).

### mRNA/LNP transfection

mRNA was transfected into HEK293T cells with the Lipofectamine 2000 (Thermo Fisher Scientific) transfection reagent according to the manufacturer’s instructions. In brief, 2 μg of mRNA was diluted in Opti-MEM medium (Gibco), coincubated with 4 μL of the transfection reagent at room temperature for 20 min, and added to cells in 12-well plates; the medium was replaced with DMEM after 4 h. In contrast, purified LNPs were added directly to cells cultured with DMEM containing 10% FBS, and the supernatant and lysate were harvested after transfection.

### Western blot

The expression of mRNA and LNPs was analyzed by western blot. Whole-cell lysates (WCLs) and supernatants were combined with loading buffer containing dithiothreitol, separated by 10% SDS-PAGE, and transferred onto a polyvinylidene difluoride (PVDF) membrane using a semidry blotting apparatus (15 V, 60 min). The membrane was blocked with nonfat milk in TBS buffer containing 0.5% Tween-20. Samples were incubated with a primary antibody (rabbit polyclonal antibodies against SARS-CoV-2 S1 at 0.1 µg/mL, Sino Biological, China) for 1.5 h and then with the secondary goat anti-rabbit IgG-HRP for 45 min. The membrane was incubated with western blot substrate (Solarbio, China), and images were captured by an ECL system (CLINX, China).

### Transmission electron microscopy analysis of LNPs and ferritin nanoparticles

The LNPs were diluted to an appropriate concentration with phosphate-buffered saline, and 10 μL of the sample was placed onto a copper grid. After 5 min, the grid was stained with 1% uranyl acetate, and transmission electron microscopy (TEM) was performed with a JEM-1400 instrument (JEDL, Japan) at an acceleration voltage of 80 kV. The ferritin nanoparticles were self-assembled by mRNA translation. In brief, mRNA was transfected into HEK293T cells, and the supernatant was harvested after 48 h. After centrifugation, dialysis, and concentration, the sample was analyzed by TEM.

### Mouse experiments

Female C57BL/6 mice aged 6–8 weeks were selected for the immunological evaluation of mRNA-RBD LNPs. Groups of mice (*n* = 10) were immunized i.m. once with a low dose (1.5 μg per mouse) or high dose (15 μg per mouse), with poly(C) LNPs serving as the placebo control. The mice were muscularly immunized with one (prime) and two (boost) doses at 2-week intervals. Spleen samples were acquired at the indicated time points after vaccination for enzyme-linked immunospot assay (ELISPOT) and intracellular cytokine staining (ICS) assays. Serum samples were collected immediately, inactivated at 56 °C for 30 min, and stored at −20 °C for subsequent use.

For virus challenge experiments, each group was comprised of female C57BL/6 mice aged 6–8 weeks (*n* = 5). Via routine immunization at a 2-week interval, the human ACE2 gene was transferred from the respiratory tract to the lungs via adenovirus vectors after 2 weeks of booster immunization. Five days later, the mice were intranasally inoculated with 1 × 10^5^ plaque-forming units (PFUs) of live SARS-CoV-2, and their body weights were monitored for 5 days. Lung tissues were harvested for viral load detection at 5 days after the challenge, and the animals were euthanized with a low dose of isoflurane.

In immunization and challenge animal experiments, the clinical statuses and food intakes of the mice were monitored and recorded daily.

### ELISA

Ninety-six-well ELISA plates (Corning, USA) were coated with the RBD protein (1 μg/mL, Sino Biological) overnight at 4 °C and blocked with 4% bull serum albumin. The serum was twofold diluted and added to each well, and the plates were incubated with goat anti-mouse IgG-HRP and developed by the addition of 100 µL of 3,3′,5′,5-tetramethylbenzidine (TMB) to each well. Finally, 100 µL of 2 mmol/L H_2_SO_4_ was added to terminate the reaction, and the light absorption of the plate was measured at 450 nm using a microplate reader (Thermo Scientific). The OD value of the highest dilution was 2.1-fold higher than that of the negative control at the same dilution and was used as the serum endpoint dilution titer. Each experiment was performed three times.

### ELISPOT

To detect specific T lymphocyte responses, the IFNγ ELISPOT assay was performed. The mice were euthanized, and their spleens were removed under aseptic conditions. Spleen cell (4 × 10^5^/well) suspensions and SARS-CoV-2 RBD protein diluents (10 μg/mL, Sino Biological, China) were added to 96-well ELISPOT plates (DAKEWE, China) precoated with IFNγ antibodies, and ConA was added as a positive control. Cells incubated without stimulation were employed as the negative control. After 18 h of incubation, the biotinylated antibody, streptavidin-HRP, and fresh substrate were added to the plates. Finally, the reaction was stopped by rinsing the plate with deionized water. The number of spots was determined using a CTL Spot Reader (CTL, USA).

### ICS assay

Antigen-specific CD4^+^ and CD8^+^ T lymphocyte immune responses were characterized by the ICS assay. In brief, mouse spleens were removed under aseptic conditions and stimulated with the RBD of the S protein (10 μg/mL, Sino Biological, China). Then, the cells were incubated with GolgiPlug (BD Biosciences, USA), incubated and stained with Fixable Dye eFluor 506, and the CD8-Percp-eFluor 710, CD3e-eFluor 450 (eBioscience, USA), CD45-APC-Cy7, and CD4-FITC (BD Biosciences, USA) surface markers. The cells were then fixed in permeabilization buffer (Thermo Fisher Scientific, USA) and stained with IFNγ-PE-eFlour 610, IL4-PE-Cyanine7, and TNFα-APC (eBiosciences, USA). All labeled lymphocytes were analyzed on a FACSAria III flow cytometer (BD Biosciences), and the data were analyzed using FlowJo V10.

### Pseudovirus neutralization assay

For pseudovirus production, the codon-optimized full-length S protein variants Alpha (B.1.1.7), Beta (B.1.351), and Delta (B.1.672) were derived from wild-type (WT) SARS-CoV-2 S protein plasmids (Table S1). The plasmid pCAGGS-S-Wuhan-Hu-1, pCAGGS-S-B.1.1.7, pCAGGS-S-B.1.351, or pCAGGS-S-B.1.617.2 was cotransfected together with psPAX2 and pLenti-GFP into HEK293T cells at a mass ratio of 1:1:1. After 48 h, the supernatant containing pseudovirus was harvested and stored at −80 °C for subsequent use. TCID_50_ was determined based on the relative luciferase activity by pseudovirus infection of HEK293T/hACE2 cells.

HEK293T/hACE2 cells were inoculated before the experiment. Starting with a 1:10 dilution, each sample was continuously diluted twice in a 96-well plate. Equal volumes of pseudovirus and variant were mixed with each diluted serum sample and incubated for 1.5 h at 37 °C. The mixture of virus-serum was added to the cells. After 72 h, the cells-supernatant mixture was collected, the firefly luciferase activity in the cells was detected by chemiluminescence, and the luciferase activity was quantified to measure the transduction efficiency. To calculate the neutralization efficiency, pseudovirus without antibody was used as a positive control. Each sample was assessed in three repeat wells. Positive values were determined to be relative luminescence unit (RLU) values that were tenfold higher than that of only the cell background. The half-maximum neutralization titer (NT_50_) value was the reciprocal of the dilution of half of the mean RLU value of the positive control.

### Live SARS-CoV-2 neutralization assay

The neutralizing activity of serum containing live SARS-CoV-2 was determined by the plaque reduction neutralization assay. Briefly, heat-inactivated serum was diluted and incubated with 100 TCID_50_ of SARS-CoV-2 at 37 °C for 1 h. The virus-serum mixture was added to preseeded Vero E6 cells and incubated for 72 h. Finally, the dilution at which cytopathic effects (CPE) appeared was recorded, and the NT_50_ value was the reciprocal of the dilution required for 50% neutralization of viral infection.

### Histopathology and fluorescence immunohistochemistry

The lung tissues were fixed in 4% paraformaldehyde for 24 h, and 5-μm-thick paraffin-embedded sections were processed for hematoxylin and eosin (H&E) staining to analyze histopathological changes in the lungs. Fluorescence immunohistochemistry (FIHC) was performed to determine the location of the virus. For FIHC, the paraffin sections were sequentially inoculated with a SARS-CoV-2-S S1 antibody (Sino Biological, China) and then with FITC-labeled goat anti-rabbit IgG; nuclei were stained with DAPI. Images were captured using a Pannoramic MIDI instrument (3D HISTECH, Hungary) and rendered using CaseViewer V2.4.0.

### Viral load measurement

The lung viral loads were determined by quantitative PCR (qRT-PCR). Briefly, mouse lungs were homogenized, and 200 μL of supernatant was extracted using a viral RNA kit (QIAGEN, Germany). Reverse transcription and TaqMan qRT-PCR were performed using a probe one-step PCR kit (TOYOBO, Japan) on an ABI Prism 7500 Real-Time PCR system. Viral loads are expressed as the viral copies/g, and the limit of detection was 100 copies/mL.

The following sequences were used:

forward primer (N): 5′-GGGGAACTTCTCCTGCTAGAAT-3′;

reverse primer (N): 5′-CAGACATTTTGCTCTCAAGCTG-3′; and

probe: 5′-FAM-TTGCTGCTGCTTGACAGATT-TAMRA-3′.

### Statistical analysis

All of the data are presented as the mean ± standard error of the mean. *P* values were determined by one-way ANOVA or Spearman rank-correlation tests. All graphs were generated with GraphPad Prism version 9.0 software.

## Supplementary information

Supplementary Materials

Data S1

## Data Availability

Further information and requests for resources and reagents should be directed to J.L. and W.L. The sequences of SARS-CoV-2 have been deposited in GenBank with the Accession codes NC_045512.2. Source data are provided with this paper.
