# Supplementary material for: The self-assembled nanoparticle-based trimeric RBD mRNA vaccine elicits robust and durable protective immunity against SARS-CoV-2 in mice
Source: Signal Transduct Target Ther. 2021 Sep 9;6:340. doi: 10.1038/s41392-021-00750-w (PMC8426336; doi:10.1038/s41392-021-00750-w)
Supplement: Supplementary file 1 — Supplementary Materials [file 41392_2021_750_MOESM1_ESM.pdf]

Supplementary Materials

The Self-assembled Nanoparticle-based Trimeric RBD mRNA Vaccine Elicits Robust and Durable Protective Immunity Against SARS-CoV-2 in Mice

Wenqiang Sun[1,2,3], Lihong He[1,4], He Zhang[1,2], Xiaodong Tian[1], Zhihua Bai[1,4], Lei Sun[1,4], Limin Yang[1], Xiaojuan Jia[1], Yuhai Bi[1,4], Tingrong Luo[3], Gong Cheng[2,6], Wenhui Fan[1*], Wenjun Liu[1,2,3,4,5*], Jing Li[1,4,7*]

[1]CAS Key Laboratory of Pathogenic Microbiology and Immunology, Institute of Microbiology, Chinese Academy of Sciences, Beijing 100101, China
[2]Institute of Infectious Diseases, Shenzhen Bay Laboratory, Shenzhen, Guangdong, 518000, China
[3]State Key Laboratory for Conservation and Utilization of Subtropical Agro-Bioresources & Laboratory of Animal Infectious Diseases, College of Animal Sciences and Veterinary Medicine, Guangxi University, Nanning 530004, Guangxi, China
[4]University of Chinese Academy of Sciences, Beijing 100049, China
[5]Institute of Microbiology, Center for Biosafety Mega-Science, Chinese Academy of Sciences, Beijing 100101, China
[6]Tsinghua-Peking Center for Life Sciences, School of Medicine, Tsinghua University, Beijing, 100084, China
[7]Lead Contact
Correspondence: J.L. (lj418@163.com), W.L. (liuwj@im.ac.cn), L.S. (sunlei362@im.ac.cn), and W.F. (fanwenhui78@aliyun.com)

This PDF file includes:

Materials and Methods
Figures S1 to S6
Tables S1

Other Supplementary Materials for this manuscript include the following:

Data S1

Materials and Methods

ELISA

Ninety-six-well ELISA plates (Corning, USA) were coated with the RBD protein (1 μg/mL, Sino Biological) overnight at 4°C and blocked with 4% bull serum albumin. The serum was twofold diluted and added to each well, and the plates were incubated with goat anti-mouse IgG-HRP and developed by the addition of 100 μL of 3,3',5',5-tetramethylbenzidine (TMB) to each well. Finally, 100 μL of 2 mmol/L H_2SO_4 was added to terminate the reaction, and the light absorption of the plate was measured at 450 nm using a microplate reader (Thermo Scientific). The OD value of the highest dilution was 2.1-fold higher than that of the negative control at the same dilution and was used as the serum endpoint dilution titer. Each experiment was performed three times.

ELISPOT

To detect specific T lymphocyte responses, the IFNγ ELISPOT assay was performed. The mice were euthanized, and their spleens were removed under aseptic conditions. Spleen cell (4×10^5/well) suspensions and SARS-CoV-2 RBD protein diluents (10 μg/mL, Sino Biological, China) were added to 96-well ELISPOT plates (DAKEWE, China) precoated with IFNγ antibodies, and ConA was added as a positive control. Cells incubated without stimulation were employed as the negative control. After 18 h of incubation, the biotinylated antibody, streptavidin-HRP, and fresh substrate were added to the plates. Finally, the reaction was stopped by rinsing the plate with deionized water. The number of spots was determined using a CTL Spot Reader (CTL, USA).

ICS assay

Antigen-specific $CD4^+$ and $CD8^+$ T lymphocyte immune responses were characterized by the ICS assay. In brief, mouse spleens were removed under aseptic conditions and stimulated with the RBD of the S protein (10 μg/mL, Sino Biological, China). Then, the cells were incubated with GolgiPlug (BD Biosciences, USA), incubated and stained with Fixable Dye eFluor 506 and the CD8-Percp-eFluor 710, CD3e-eFluor 450 (eBioscience, USA), CD45-APC-Cy7 and CD4-FITC (BD Biosciences, USA) surface markers. The cells were then fixed in permeabilization buffer (Thermo Fisher Scientific, USA) and stained with IFNγ-PE-eFlour 610, IL4-PE-Cyanine7 and TNFα-APC (eBiosciences, USA). All labeled lymphocytes were analyzed

on a FACSAria III flow cytometer (BD Biosciences), and the data were analyzed using FlowJo V10.

Pseudovirus neutralization assay

For pseudovirus production, the codon-optimized full-length S protein variants Alpha (B.1.1.7), Beta (B.1.351) and Delta (B.1.672) were derived from wild-type (WT) SARS-CoV-2 S protein plasmids (Table S1). The plasmid pCAGGS-S-Wuhan-Hu-1, pCAGGS-S-B.1.1.7, pCAGGS-S-B.1.351 or pCAGGS-S-B.1.617.2 was cotransfected together with psPAX2 and pLenti-GFP into HEK293T cells at a mass ratio of 1:1:1. After 48 h, the supernatant containing pseudovirus was harvested and stored at −80 °C for subsequent use. $TCID_{50}$ was determined based on the relative luciferase activity by pseudovirus infection of HEK293T/hACE2 cells.

HEK293T/hACE2 cells were inoculated before the experiment. Starting with a 1:10 dilution, each sample was continuously diluted twice in a 96-well plate. Equal volumes of pseudovirus and variant were mixed with each diluted serum sample and incubated for 1.5 h at 37 °C. The mixture of virus-serum was added to the cells. After 72 h, the cells-supernatant mixture was collected, the firefly luciferase activity in the cells was detected by chemiluminescence, and the luciferase activity was quantified to measure the transduction efficiency. To calculate the neutralization efficiency, pseudovirus without antibody was used as a positive control. Each sample was assessed in three repeat wells. Positive values were determined to be relative luminescence unit (RLU) values that were tenfold higher than that of only the cell background. The half-maximum neutralization titer (NT_{50}) value was the reciprocal of the dilution of half of the mean RLU value of the positive control.

Co-immunoprecipitation (Co-IP)

TF-RBD-flag, TF-RBD-1mut-myc, and TF-RBD-3mut-strep mRNAs were co-transfected into HEK293T cells by Lipofectamine 2000 (Thermo Fisher Scientific) transfection reagent. After 48 h, collected the cell lysate and use ANTI-FLAG M2 agarose beads (Sigma) to enrich the binding proteins. The enriched proteins were analyzed by Western blot.

Figures S1 to S6

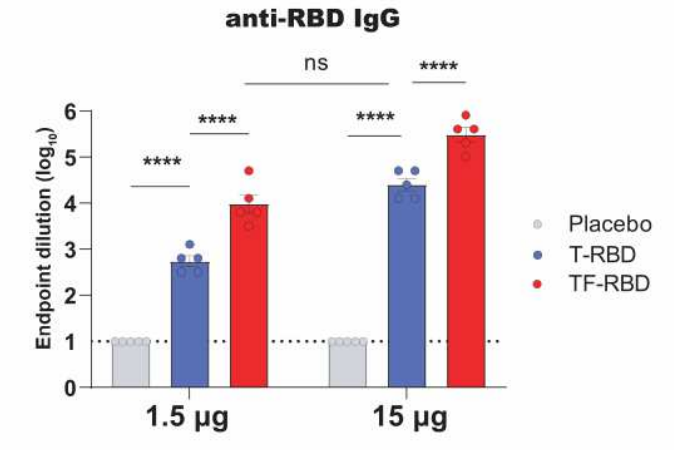

Figure S1. The RBD-specific IgG titer of SARS-CoV-2 as determined by ELISA. Related to Figure 3. Serum samples were collected after 14 days of booster immunization. The dotted horizontal lines indicate the limits of quantification for ELISA. The data are shown as the mean ± SEM. P values were determined by one-way ANOVA (ns, $p > 0.05$; * $p < 0.05$; ** $p < 0.01$; *** $p < 0.001$; **** $p < 0.0001$).

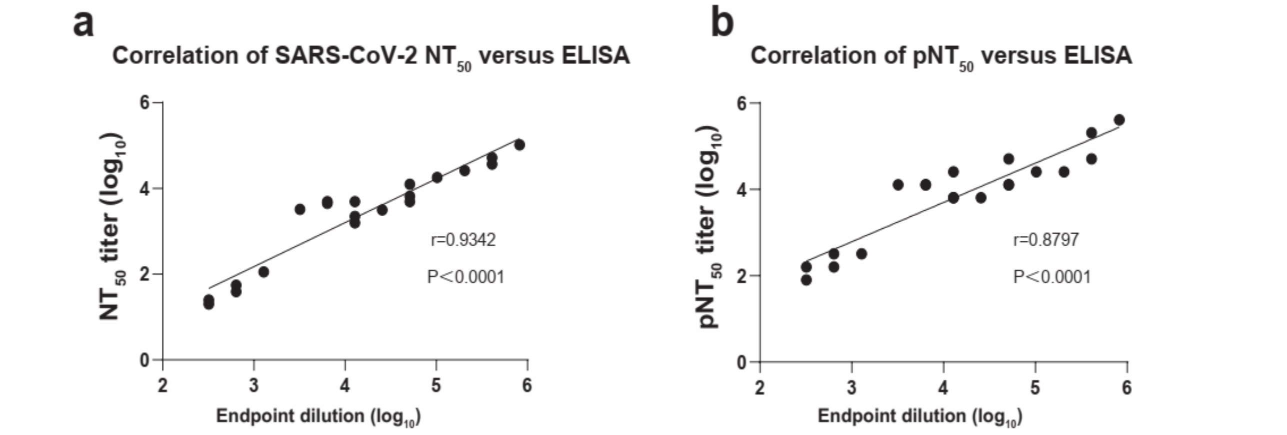

Figure S2. Neutralizing activity of serum samples in relation to the ELISA titers. Related to Figure 3. (a, b) Correlation analyses of the ELISA titers on the x-axis and the neutralization titers in a live SARS-CoV-2 neutralization assay (a) and pseudovirus neutralization assay (b) on the y-axis. The correlation analysis was based on the ELISA (n=20) and neutralization assay (n=20) data in the low- and high-dose T-RBD and TF-RBD groups. The r and P values were determined by Spearman rank-correlation tests.

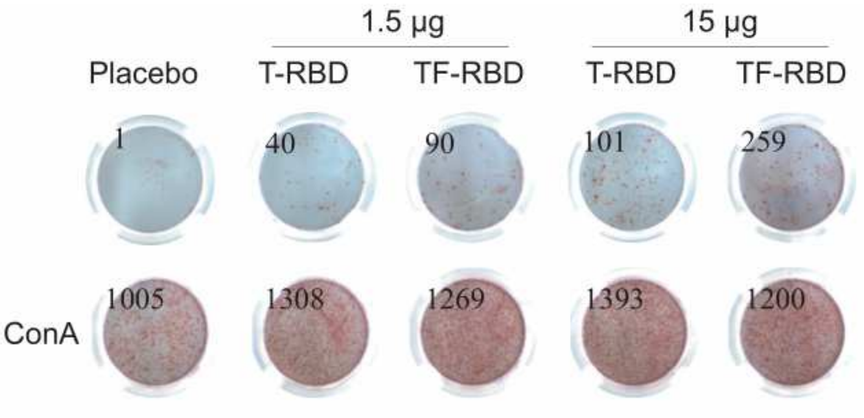

Figure S3. Protein-specific T cell ELISPOT assay results. Related to Figure 3. Spleen cells (2×10^5) were stimulated with the S RBD protein at a final concentration of 10 μg/mL. Concanavalin A (ConA) was applied as a positive stimulus. The ELSPOT images were captured by an automatic ELISPOT reader, and the number of spots was analyzed by ImmunoCapture 6.5.0 software.

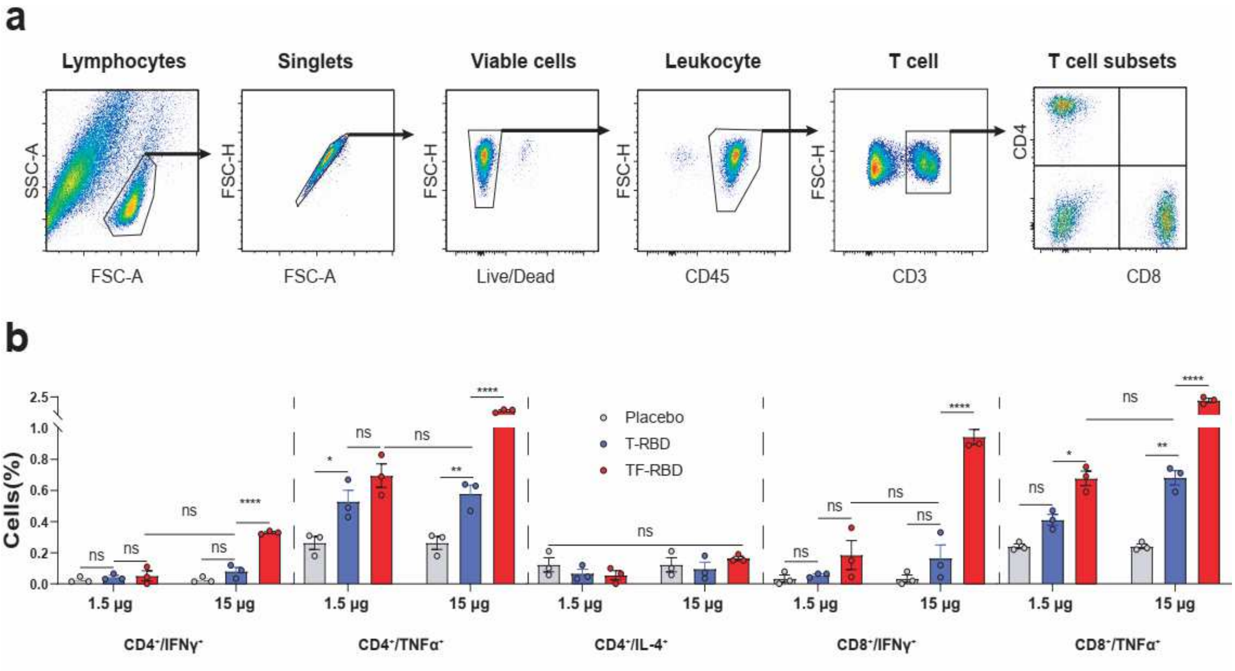

Figure S4. Conduction of protein-specific ICS assays. Related to Figure 3. (a) Gating strategy for intracellular staining flow cytometry. (b) Schematic of the ICS assays to measure the proportions of IFNγ and TNFα in $CD4^+$ and $CD8^+$ protein-specific T cells. The data are shown as the mean ± SEM. P values were determined by one-way ANOVA (ns, $p > 0.05$; * $p < 0.05$; ** $p < 0.01$; *** $p < 0.001$; **** $p < 0.0001$).

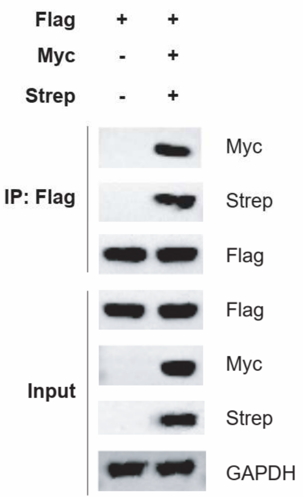

Figure S5. The Co-IP assay was used to verify that TF-RBD-Flag, TF-RBD-1mut-myc and TF-RBD-3mut-strep could be assembled into nanoparticles together. Related to Figure 5. TF-RBD-Flag, TF-RBD-1mut-myc and TF-RBD-3mut-strep mRNA were co-transfected into HEK293T cells, the proteins were enriched with an-flag beads and the results were analyzed by WB.

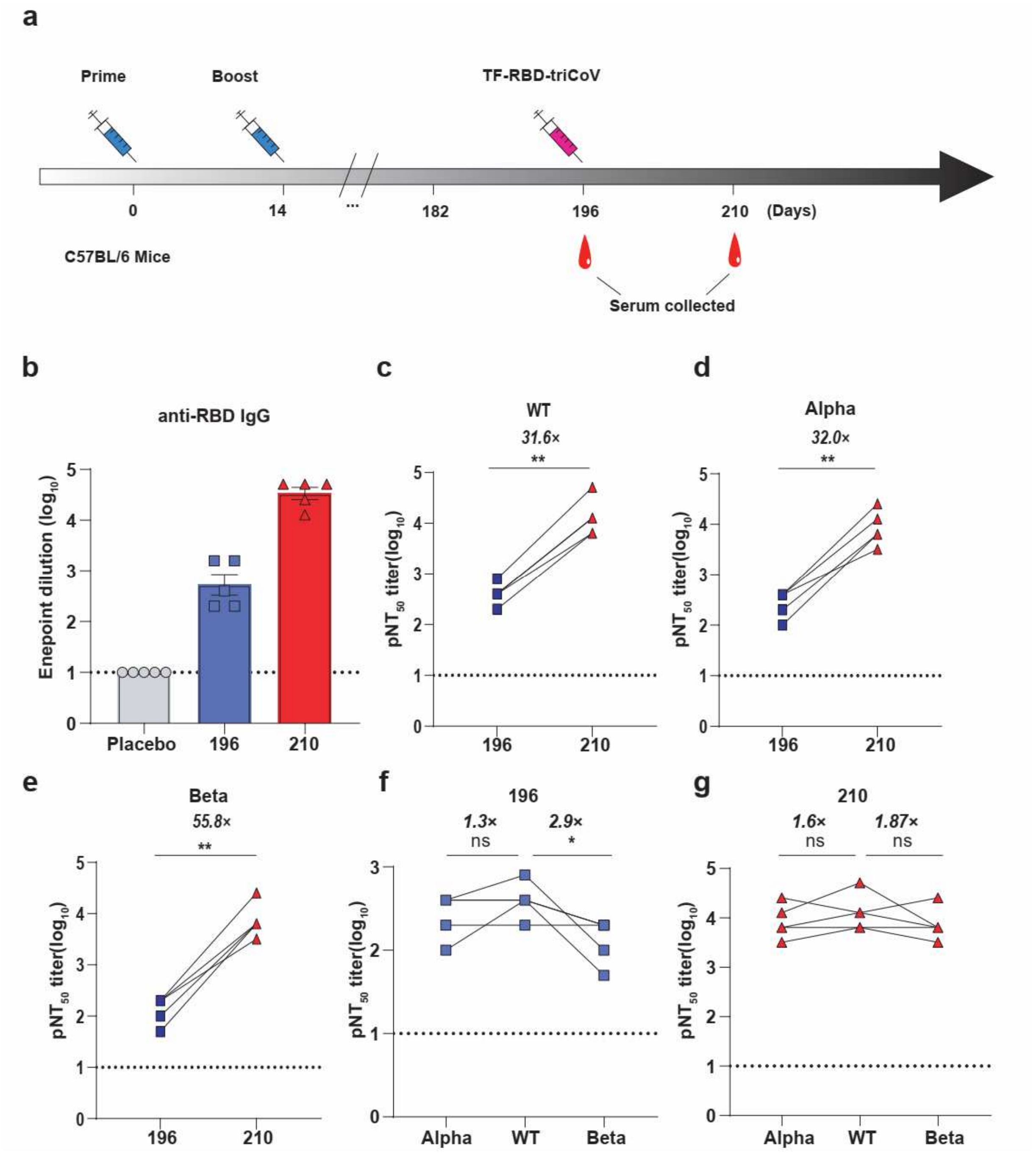

Figure S6. Humoral immunity evaluation of a boost with TF-RBD-triCoV after two doses of TF-RBD. Related to Figure 5. (a) Immune procedures for evaluating the humoral immunity of TF-RBD-triCoV. The mice (n = 5) pre-immunized with two doses of TF-RBD (1.5 μg) were vaccinated with a 1.5 μg dose of TF-RBD-triCoV at day 196. Blood collection was performed at the indicated time points. A placebo formulation was given as the control. (b) The RBD-specific IgG titer of SARS-CoV-2 was revealed by ELISA. (c-e) The pNT50 comparison between day

196 and 210 of vaccination against WT (c), Alpha (d) and Beta (e) pseudoviruses. (f, g) the cross-neutralization titers at day 196 (g) and 210 (g) against the different pseudoviruses. The dotted horizontal lines indicate the limits of quantification for ELISA and pNAb titers. The data are shown as the mean ± SEM. P values were determined by one-way ANOVA (ns, $p > 0.05$; * $p < 0.05$; ** $p < 0.01$; *** $p < 0.001$; **** $p < 0.0001$).

Table S1. S proteins of SARS-CoV-2 variant for pseudoviral neutralization assay

Variant CoVs	Amino acid mutation site of Wuhan-Hu-1 S proteins
Alpha (B.1.1.7)	ΔH69-V70, ΔY144, N501Y, A570D, D614G, P681H, T716I, S982A, D1118H
Beta (B.1.351)	L18F, D80A, D215G, Δ242-244, R246I, K417N, E484K, N501Y, D614G, A701V
Delta (B.1.617.2)	T19R, Δ157-158, L452R, T478K, D614G, P681R, D950N

Data S1.

Amino acid sequence of TF-RBD

MDAMKRGLCCVLLLCGAVFVSPSRVQPTESIVRFPNITNLCPFGEVFNATRFASVYAWN
RKRISNCVADYSVLYNSASFSTFKCYGVSPTKLNDLCFTNVYADSFVIRGDEVRQIAPGQ
TGKIADYNYKLPDDFTGCVIAWNSNNLDSKVGGNYNYLYRLFRKSNLKPFERDISTEIY
QAGSTPCNGVEGFNCYFPLQSYGFQPTNGVGYQPYRVVVLSFELLHAPATVCGPKKSTN
LVKNKCVNFGSGGYIPEAPRDGQAYVRKDGEWVLLSTFLGGGGSGGGGSLSKDIIKLLN
EQVNKEMNSSNLYMSMSSWCYTHSLDGAGLFLFDHAAEEYEHAKKLIIFLNENNVPVQ
LTSISAPEHKFEGLTQIFQKAYEHEQHISESINNIVDHAIKSKDHATFNFLQWYVAEQHEE
EVLFKDILDKIELIGNENHGLYLADQYVKGIAKSRKS
