# Supplementary material for: The self-assembled nanoparticle-based trimeric RBD mRNA vaccine elicits robust and durable protective immunity against SARS-CoV-2 in mice
Source: Signal Transduct Target Ther. 2021 Sep 9;6:340. doi: 10.1038/s41392-021-00750-w (PMC8426336; doi:10.1038/s41392-021-00750-w)
Supplement: Supplementary file 2 — Data S1 [file 41392_2021_750_MOESM2_ESM.docx]

**Amino acid sequence of TF-RBD：**MDAMKRGLCCVLLLCGAVFVSPSRVQPTESIVRFPNITNLCPFGEVFNATRFASVYAWNRKRISNCVADYSVLYNSASFSTFKCYGVSPTKLNDLCFTNVYADSFVIRGDEVRQIAPGQTGKIADYNYKLPDDFTGCVIAWNSNNLDSKVGGNYNYLYRLFRKSNLKPFERDISTEIYQAGSTPCNGVEGFNCYFPLQSYGFQPTNGVGYQPYRVVVLSFELLHAPATVCGPKKSTNLVKNKCVNFGSGGYIPEAPRDGQAYVRKDGEWVLLSTFLGGGGSGGGGSLSKDIIKLLNEQVNKEMNSSNLYMSMSSWCYTHSLDGAGLFLFDHAAEEYEHAKKLIIFLNENNVPVQLTSISAPEHKFEGLTQIFQKAYEHEQHISESINNIVDHAIKSKDHATFNFLQWYVAEQHEEEVLFKDILDKIELIGNENHGLYLADQYVKGIAKSRKS
